# Non-apoptotic roles of apoptotic proteases: new tricks for an old dog

**DOI:** 10.1098/rsob.200130

**Published:** 2020-08-19

**Authors:** Tin Tin Su

**Affiliations:** 1 Department of Molecular, Cellular and Developmental Biology. University of Colorado, 347 UCB, Boulder, CO 80309-0347, USA; 2 Molecular and Cellular Oncology Program, University of Colorado Comprehensive Cancer Center, Anschutz Medical Campus, 13001 E. 17th Pl., Aurora, CO 80045, USA

**Keywords:** apoptosis, caspase, plasticity

## Abstract

This Open Question article highlights current advances in the study of non-apoptotic roles of apoptotic proteins. Apoptosis is a highly regulated and energy-requiring process in which cells actively kill themselves. Apoptosis helps remove extra cells to sculpt organs during embryo development and culls damaged cells throughout the body. Apoptosis relies on evolutionarily conserved proteins that include a family of proteases called caspases. Caspases activity has long been considered a hallmark of apoptosis. Yet an emerging body of literature indicates that caspase activity is required for a number of non-lethal processes that range from sculpting cells, removing protein aggregates, changing cell identity during differentiation or de-differentiation, and rebuilding tissues. Failure in each of these processes is associated with human disease. This article is not meant to be an exhaustive review but an introduction to the subject for an educated public, with caspases as a gateway example. I propose that it is time to explore non-apoptotic roles of caspases and other apoptotic proteins, in order to better understand their non-apoptosis function and to leverage new knowledge into new therapies.

## 1. Introduction: demolish or remodel?

**Figure 1.**
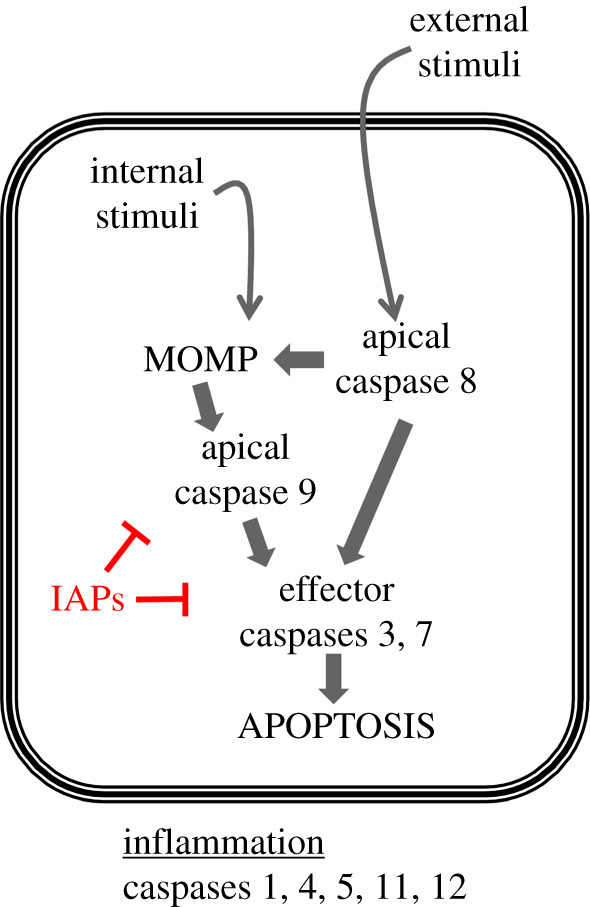
Induction of apoptosis by caspases. Internal or external stimuli result in mitochondrial outer membrane permeabilization (MOMP) and activation of apical caspases 8 or 9. These in turn activate effector caspases 3 and 7 to induce apoptosis. Inhibitor of apoptosis proteins (IAPs) temper caspase activity. A different subset of caspases plays a role in inflammation and are not discussed here.

Apoptosis is a process in which a cell commits suicide. It can last as long as a day and proceeds through recognizable steps. Apoptosis requires caspases, a family of aspartate-directed proteases, that are made as inactive proenzymes (figure 1, for review, [1]). Internal damage or external death-inducing stimuli such as Fas and TNF ligands result in mitochondrial outer membrane permeabilization (MOMP). In most organisms, the release of cytochrome C from porous mitochondria into the cytoplasm results in cleavage and activation of apical caspases (reviewed in [2]). Apical caspases are also activated in response to death stimuli in *Drosophila* but the requirement for MOMP remains controversial in this organism.

Active apical caspases cleave to activate effector caspases, which then destroy non-caspase proteins to bring about apoptosis. Activation of caspases was long thought to be a point-of-no-return; in fact, cleaved caspases are used widely as surrogate markers for apoptosis. But hints of a more complicated picture existed as far back as three decades ago when characteristic features of apoptosis were recognized in terminally differentiating cells that were very much alive [3]. Many studies since have documented that cells in diverse organisms can survive non-lethal caspase activation that, instead of killing a cell, helps change its interior [4–8]. If we think of a cell as a house, apoptosis is to demolish it completely. One could instead remodel, with results ranging from the removal of parts of a cell to completely altering its identity, much like turning a Victorian into a Tudor. And it is the remodelling of the cells using caspase activity that is attracting attention. We see evidence of it in cells of central and peripheral nervous systems, blood and muscle, and in sperm and stem cells. We see evidence of it in a variety of organisms that include nematode, fruit fly, chick and mice. Caspase-dependent non-apoptotic processes have implications in cancer, neurodegeneration, wound repair and regeneration. Yet many questions remain such as how caspase activity is controlled to result in cellular remodelling instead of death and what the relevant substrates for the non-lethal roles of caspases are. It is by studying non-apoptotic roles of these conserved enzymes that we will reach a more complete understanding of the natural world and also hope to identify new drug targets for a variety of human diseases. The following paragraphs use select examples to illustrate the diversity of functions provided by non-lethal caspase activity and propound the potential benefits of their further study.

I wish to distinguish examples discussed here from a process called anastasis (reviewed in [6,9–12]). Anastasis describes a cell that returns to life from the brink of apoptotic death. Anastasis has been observed in cells exposed to a sub-lethal dose of apoptotic stimuli (for example, [13]) or if the apoptotic stimulus was withdrawn before apoptosis could be completed (for example, [14–16]). A cell that went through anastasis suffers abnormalities such as chromosomal translocations and an increased propensity for oncogenic transformation, which occur in a caspase-dependent manner [13,15]. The role of caspases in anastasis, however, is in line with their role in apoptosis. The examples discussed here focus instead on non-apoptotic roles of caspases, often with little information on how close cells came to apoptosis in each case.

## 2. When apoptotic caspases sculpt individual cells

**Figure 2.**
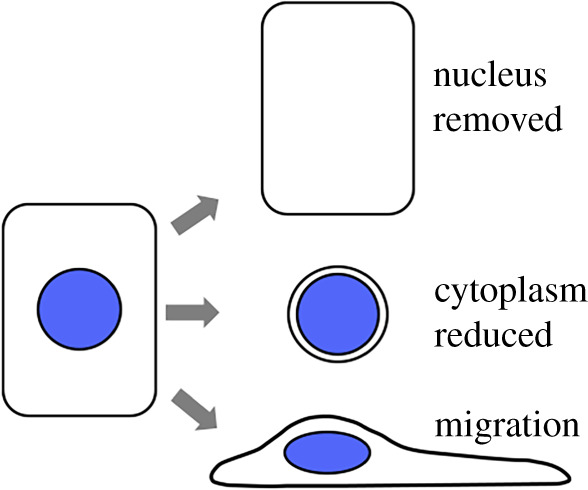
Three ways to remodel a cell: removal of the nucleus (top), reduction of the cytoplasm (middle), or migration (bottom).

All cells of our body descend from pluripotent cells that specialized to become different cell types. Specialization of cells accompanies the remodelling of cellular contents, and caspase activity is required in several such instances (figure 2).

### 2.1. Removal of the nucleus

An extreme form of cellular remodelling occurs when organelles inside a cell, including the entire nucleus, are removed. This helps to clarify lens cells for light transmission and make red blood cells (RBCs) more efficient oxygen carriers [17,18]. Differentiating lens fibres of chick embryos express caspases 1–4 (all that were examined). Inhibition of caspase activity with peptide inhibitors prevented nuclear degeneration in cultured lens fibres, suggesting that this process is caspase dependent [17]. Likewise, RBC precursors show nuclear and chromatin condensation, the loss of organelles and ultimately the loss of the nucleus. These changes were accompanied by the activation of effector caspases and were blocked by the addition of a caspase inhibitor to culture [18]. Caspase substrates in RBC differentiation include nuclear proteins lamin B and acinus. Caspase 3 activation was lower during RBC differentiation than if the same cells are exposed to an apoptotic stimulus, suggesting that quantitative differences in caspase activity may explain why RBC precursors survive and differentiate instead of die.

### 2.2. Downsizing the cytoplasm

An alternate form of extreme remodelling happens when cells keep the nucleus but reduce the cytoplasm. Drice (*Drosophila* caspase 3) facilitates this change during the final step of sperm differentiation [19]. Inhibiting caspase activity with ectopic expression of viral caspase inhibitor p35 prevented cytoplasmic elimination and produced sterile males, illustrating the importance of caspase activity in sperm development. Caspase 3 activity appears to be tempered by an IAP-like protein dBRUCE (figure 1 for where IAPs function) because, without it, sperm nuclei degenerated much like in apoptosis.

In some cases of remodelling, the cytoplasmic loss is partial and occurs through spatially restricted caspase activity. One such example is neuronal pruning, the process in which axons or dendrites are selectively removed in order to establish the correct circuitry. Neuronal pruning in mice requires caspases 3 and 9 whose activity is kept away from the cell body and restricted to axons to be pruned, by proteasome activity and localized expression of an IAP protein [20]. Spatially restricted caspase activation is responsible also for the pruning of sensory neuron dendrites in *Drosophila* where global caspase activation resulted in cell death [21]. Pruning allows for neuronal plasticity, which is important for learning and memory. This can explain the importance of caspases to songbirds [22]. In Zebra finch auditory forebrain, the expression of transcription factor *zenk* increases after hearing a new song but the increase is dampened once the song has been learned in a phenomenon called ‘habituation'. Caspase 3 is active in the same brain region after hearing a song, and infusion of a caspase 3 inhibitor decreased habituation as if the birds had not heard the song before.

### 2.3. Cell migration

Dynamic remodelling of the cell without the loss of cellular contents occurs during cell migration. Both apical and effector caspases have a role in this process. Knocking out effector caspase 3 from colorectal cancer cells reduced their motility in *in vitro* culture and reduced their metastatic capability in mouse xenografts [23]. Relevant substrates remain to be identified. Apical caspase 8 is also required for motility in mouse embryonic fibroblasts and human cancer cells but, surprisingly, its catalytic activity is not necessary [24,25]. Instead, caspase 8 localizes to the lamella (the leading part) of migrating cancer cells where it is proposed to help organize protein complexes needed for migration [26]. In counter examples, caspases INHIBIT cell migration in *Drosophila*. During dorsal closure in the pupa, a process in which two single-cell layers migrate towards each other, caspase activity but not apoptosis was detected in migrating cells. Inhibiting apical or effector caspase activity accelerated migration, suggesting that caspase activity normally plays an inhibitory role to modulate the speed of migrating cells [27]. Likewise, mutations in effector caspases increased the migratory behaviour of irradiated larval cells, suggesting an inhibitory role [28]. Relevant substrates remain to be identified in these cases.

## 3. When caspases change cellular identity

**Figure 3.**
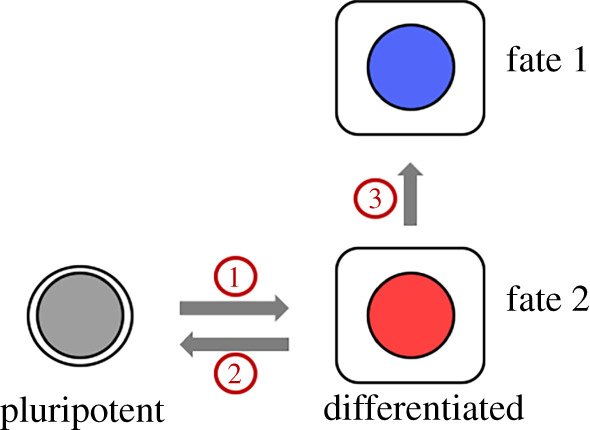
A pluripotent cell is capable of differentiation into different fates. Apoptotic caspases are needed for differentiation (1), de-differentiation (2) and switching between different fates by differentiated cells (3).

Cell remodelling is profound when a cell with a new identity and function emerges from a stem cell (figure 3, ‘1'), when a differentiated cell returns into a pluripotent state (figure 3, ‘2'), or when one differentiated cell turns into another (figure 3, ‘3'). The following examples illustrate how caspases promote these changes. The emerging view is that caspases degrade proteins associate with the original identify, allowing the new identity to take hold.

### 3.1. Differentiation into a specialized cell fate

Caspase 3^−/−^ mice exhibits an unexpected phenotype, namely reduced skeletal muscle mass. Isolated myoblasts from such mice failed to differentiate into myotubes in culture, indicating a requirement for caspase activity in a late step in muscle differentiation [29]. Since then, many instances of cellular differentiation, in either normal development or regeneration, show accompanying caspase activity without concomitant apoptosis (for example, see [29–31]; reviewed in [32]). In some such cases, functionally relevant caspase substrates, typically pluripotent factors, have been identified. These include NANOG in mouse embryonic stem cells where a caspase-resistant version of NANOG prevented differentiation [33]; PAX7 in muscle stem cells [34]; *C. elegans* LIN-28 whose cleavage is required for the completion of the L1/L2 larval state [35,36]; MST1 kinase in muscle terminal differentiation where a shortened MST1 that mimics the cleaved product rescued differentiation in caspase 3^−/−^ cells [29,37] and Sgg (*Drosophila* GSK3 kinase) whose cleavage and activation control Wingless (*Drosophila* Wnt) signalling to limit the number of larval sensory organ precursors [38]. In the case of Sgg, the regulation of IAP turnover through a *Drosophila* IKK-like kinase tempers caspase activity to provide a developmental role [31,39]. But in most of these cases, how caspase activity is controlled to inhibit apoptosis yet meet the need for differentiation remains to be understood.

### 3.2. The reverse: promoting stem-ness

Our ability to use a handful of transcription factors to turn differentiated cells into pluripotent cells has revolutionized the field of stem cell biology. One of these, Oct-4, activates caspase 3 in the generation of iPSC from human fibroblasts. More important, inhibition of caspase 3 or its activator caspase 8 blocked iPSC induction partially (caspase 3) or completely (caspase 8) [40]. Tumour suppressor and cell cycle inhibitor Rb was identified as a relevant substrate in this case but others are likely involved. In a variation of this phenomenon, effector caspases are found to sustain the stem-ness of human cancer cells [41]. Knockdown of caspase 3 in patient-derived glioma cells, for example, reduced their ability to form tumour spheres *in vitro*, a measure of stem-ness, and reduced new tumour formation in xenografts. The link between caspases and tumorigenicity is proposed to be through induction of a low level of DNA breaks that result in the opening of chromatin to provide access to transformation factors.

### 3.3. Going sideways: switching cell types

Imaginal discs of *Drosophila* larvae are precursors of adult structures that display an incredible ability to regenerate without dedicated stem cells. Recent studies using lineage tracing document a switch of one cell type to another during regeneration [42–44]. Specifically, when the pouch region of the wing imaginal disc was ablated by the tissue-specific expression of a pro-apoptotic gene, nearby hinge cells lose hinge-specific gene expression, translocate into the pouch, express pouch markers and help regenerate the pouch [42]. In our studies using X-ray doses that kill about half of the cells in the wing disc, the hinge was found to be protected from X-ray-induced apoptosis [43]. During regeneration, X-ray-resistant hinge cells lose hinge-specific gene expression, translocate into the pouch that suffers more X-ray-induced apoptosis, express pouch markers and participate in regeneration of the latter, much like in the genetic-ablation model [43]. Inhibition of apical or effector caspases within the hinge during X-ray-induced regeneration blocked both fate change and translocation, demonstrating a cell autonomous requirement for caspase activity [44]. Because irradiated hinge cells do not die, the requirement for caspases may reflect a non-apoptotic role in cell fate plasticity. Relevant substrates remain to be identified.

## 4. When caspases clean up the cell instead of killing it

The genome of budding yeast *Saccharomyces cerevisiae* encodes a metacaspase Yca1. Metacaspases are caspase-like proteases from plants, fungi and protozoa that show structural resemblance to caspases. Metacaspases have a cystine in the active site like caspases but show different substrate specificity, cleaving after Arg or Lys instead of an Asp. *S. cerevisiae* undergoes cell death with features similar to those in animal apoptosis such as DNA fragmentation and externalization of membrane lipid phosphatidylserine, but can do so without Yca1 [45–47]. Proteomic analysis of yeast cells lacking Yca1 showed unexpected effects: an increase in stress-response proteins and the presence of insoluble protein aggregates in the cytoplasm [48,49]. Yeast cells lacking Yca1 have a reduced replicative life span [50], suggesting a role for yeast caspase-like proteins in clearing harmful protein aggregates and preventing cellular ageing.

## 5. When apoptotic proteins help rebuild tissues

Tissue homeostasis requires that dead cells are replaced by new cells. In organisms ranging from *Hydra* to mice, apoptotic caspase activity unleashes mitogenic signalling cascades, the exact components of which differ between organisms or among different cells of the same organism. Despite these differences, a common outcome is non-cell autonomous promotion of cell proliferation, which helps rebuild tissues and heal wounds. In *Drosophila* larvae, this response is known as apoptosis-induced proliferation (AiP) ([51–54]; reviewed in [55,56]). Apical caspase Dronc acts together with JNK signalling to produce secreted mitogenic signal Wg (*Drosophila* Wnt1). Localized activation of Dronc increases extracellular reactive oxygen species (ROS) that provides at least two functions: to recruit macrophages that secrete JNK ligand Eiger to sustain mitogen production [57,58] and to activate regenerative JAK/STAT signalling through p38 [59]. The role of Dronc in AiP is distinct from its role in activating effector caspases for apoptosis because AiP still occurs when the latter was inhibited.

A similar response in mice is known as ‘phoenix rising' where effector caspases 3 and 7 in mice cleave and activate calcium-independent phospholipase A2 to result in the generation and release of prostaglandin E, a known promoter of cell proliferation [60,61]. Caspase 3 in irradiated dying tumour cells promotes angiogenesis through the production of growth factor VEGF [62,63]. The role of effector caspases in promoting non-autonomous proliferation is relevant to tumour biology such that mouse mutants lacking caspase 3 show poor tumour re-growth after irradiation [60]. An AiP like process operates in plants where a metacaspase cleaves a peptide signalling molecule to elicit a wound healing response [64].

## 6. Why study non-apoptosis roles of apoptosis proteins?

Sections 2 to 5 illustrate how non-apoptotic functions of caspases affect four biological phenomena: sculpting of individual cells, changing cell identity, cleansing the cells of unwanted proteins and rebuilding tissues. Understanding these phenomena is fundamental to understanding not only the natural world but also to understanding and ultimately treating diseases. A clear illustration of the need to understand both apoptotic and non-apoptotic roles of caspase is the finding that in mouse embryonic fibroblasts, low caspase 3 activity, for example, from exposure to low levels of chemotherapeutic drug cisplatin, cleaved p120 RasGAP to release a fragment capable of survival signalling [65,66]. At higher caspase 3 activity, this fragment was cleaved further and apoptosis ensued. Thus, low caspase 3 activity was pro-survival while high caspase 3 activity was pro-death. Successful therapy of cancer often relies on the eradication of cancer cells by the induction of apoptosis. But the failure to induce sufficient caspase activity could produce cells with low caspase activity that not only survived but, as discussed in previous sections, were more migratory or stem cell-like. The result could be metastatic cells that could seed new tumour growth at distant sites, exactly the opposite of the desired outcome.

We are appreciating more and more how non-apoptotic caspase activity underlies biological phenomena in many organisms and cell types. In fact, the removal of certain proteins by non-apoptotic caspase activity should be viewed as an additional layer of post-translational modification that is imposed on the transcriptome and the proteome. With this understanding comes the potential to manipulate caspase activity towards a desired outcome. For example, RBC counts can be boosted with Epo but the increase in erythrocyte differentiation with the drug is only about 25%. The success rate of inducing iPSCs is in single digits at present. Could these efficiencies be increased with a non-lethal dose of caspase activity? Could we likewise exploit the role of metacaspases and possibly caspases in protein homeostasis to remove pathogenic protein aggregates in neuro- and muscular degeneration? Could we use non-lethal doses of caspase activity to boost learning and memory as in the case of songbirds?

## 7. Conclusion

Many other proteins besides caspases function in apoptosis and several are now known to have non-apoptotic roles. Bcl-2 family of proteins reside on the mitochondria outer membrane and regulate MOMP (figure 1), but are now known to regulate other mitochondrial functions as well as processes in the endoplasmic reticulum and other organelles [67–70]. The recognition that Bcl-2 promotes mitochondrial glycolysis to increase the survival of leukaemia stem cells led to the addition of a Bcl-2 inhibitor in the treatment regime for acute myeloid leukaemia [71]. Extremely positive results in patient survival led to accelerated FDA approval in 2018, illustrating how a better understanding of non-apoptotic roles of apoptotic proteins could lead to improved treatments. Therapeutic uses of caspase inhibitors have been explored in preclinical and clinical trials, but these either target caspases involved in inflammation and not apoptosis such as caspase 1, or aim to curb apoptotic induction by apoptotic caspases [72]. None to my knowledge exploits the non-apoptotic role of apoptotic caspases towards a therapeutic goal, yet many opportunities to do so exist in oncology, stem cell biology, regeneration and neurobiology as illustrated by examples herein. It is my hope that more of such opportunities become imminent as we study non-apoptotic roles of caspases and other apoptotic proteins.

## Data accessibility

This article has no additional data.
